# Reduced Expression of *BjRCE1* Gene Modulated by Nuclear-Cytoplasmic Incompatibility Alters Auxin Response in Cytoplasmic Male-Sterile *Brassica juncea*

**DOI:** 10.1371/journal.pone.0038821

**Published:** 2012-06-18

**Authors:** Xiaodong Yang, Xunyan Liu, Wenhui Lv, Lu Li, Qianqian Shi, Jinghua Yang, Mingfang Zhang

**Affiliations:** 1 Laboratory of Germplasm Innovation and Molecular Breeding, Institute of Vegetable Science, Zhejiang University, Hangzhou, People’s Republic of China; 2 Key Laboratory of Horticultural Plant Growth, Development & Quality Improvement, Ministry of Agriculture, Hangzhou, People’s Republic of China; 3 College of Life and Environment Sciences, Hangzhou Normal University, Hangzhou, People’s Republic of China; Nanjing Agricultural University, China

## Abstract

The signal from organelle to nucleus, namely retrograde regulation of nuclear gene expression, was largely unknown. Due to the nuclear-cytoplasmic incompatibility in cytoplasmic male-sterile (CMS) plants, we employed CMS *Brassica juncea* to investigate the retrograde regulation of nuclear gene expression in this study. We studied how reduced *BjRCE1* gene expression caused by the nuclear-cytoplasmic incompatibility altered the auxin response in CMS of *B. juncea*. We isolated the *BjRCE1* gene that was located in the nucleus from *B. juncea*. Over-expression of *BjRCE1* enhanced auxin response in transgenic *Arabidopsis*. The expression of *BjRCE1* was significantly reduced in CMS compared with its maintainer fertile (MF) line of *B. juncea*. There were fewer lateral roots in CMS than MF under normal and treatment of indole-3-acetic acid (IAA) conditions. Expression patterns of several auxin-related genes together with their phenotypes indicated a reduced auxin response in CMS compared to MF. The phenotypes of auxin response and auxin-related gene expression pattern could be mimicked by inhibiting mitochondrial function in MF. Taken together, we proposed reduced expression of *BjRCE1* gene modulated by nuclear-cytoplasmic incompatibility alters auxin response in CMS *B. juncea*. This may be an important mechanism of retrograde regulation of nuclear gene expression in plants.

## Introduction

In plant cells, mitochondria and chloroplast are semi-autonomous organelles that encode some genetic information, with the majority being derived and imported from the nucleus. Thus, there is wide inter-organellar communication between mitochondria and the nucleus. Over past years, there has been increasing attention paid to studies of signals from the nucleus to organelles, termed ‘anterograde regulation’ due to the predominant role of the nucleus in the cell, which has mainly focused on pentatricopeptide repeat (PPR) proteins that regulate RNA editing in mitochondria and chloroplast and the male fertile restorer (*Rf*) gene in CMS lines [1], [2], [3]. In contrast, organelles are also engaged in organelle-to-nucleus signals, termed ‘retrograde regulation’ that tune fork in nuclear gene expression, and are involved in responses to multiple stresses, and in growth and development [4], [5], [6]. Mitochondrial retrograde regulation (MRR) of nuclear gene expression was first investigated in yeast [7] and has been well described in yeasts and mammals (reviewed by [8]). Among the MRR pathways, the RTG (retrograde) pathway has been mostly studied in yeast, of which nuclear target gene (*CIT2*) has been identified, as well as key proteins of signal transduction, e.g. Rtg1, Rtg2 and Rtg3 [8]. However, MRR of nuclear gene expression is poorly understood in plants. Several reviews have predicted similar and conserved MRR pathways for both yeast and mammals [8], [9], [10], [11], [12]. In many cases, mutations in mitochondria cause embryo lethality due to the mitochondrial function of providing most of the cell’s energy. In plant, plastid retrograde regulation (PRR) was relatively well described, in which the GUN1 gene integrated the multiple indicators in plastid and led to ABI4-mediated the repression of nuclear gene expression [6].

**Figure 1:**
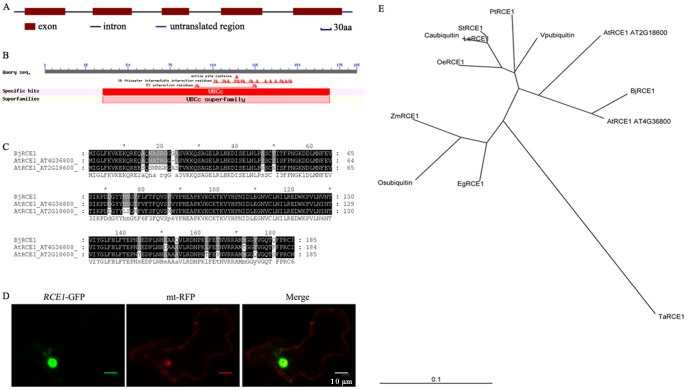
The characterization of *RCE1* gene from *Brassica juncea*. A, Genomic structure of *RCE1* gene from *Brassica juncea*. B, Conserved domain and ubiquitin interaction sites of *RCE1* gene from *Brassica juncea*. C, Alignment of *RCE1* gene from *Brassica juncea* and its orthologous from *Arabidopsis*. D, Sub-cellular localization of *RCE1* gene from *Brassica juncea*. Scale bar = 10 µm. E, Phylogenetic tree of RCE1, RCE1 amino acid sequences are from NCBI database.

The CMS system is caused by mitochondrial mutation with abundant simultaneous variant traits in plants. To date, CMS has been observed in >150 plant species and widely used in heterosis [13], [14]. In most cases, it is known to be triggered by mitochondria, usually due to novel open reading frames (*orfs*) resulting from rearrangements of mitochondrial genomes, meanwhile, for many CMS systems developed from distant hybridization and backcrossing also showed the nuclear-cytoplasmic incompatibility, which suggested not only mitochondria but also chloroplast were involved in the communication between organelles and nucleus [1], [2], [3]. Therefore, the CMS system is an ideal model to study retrograde regulation of nuclear gene expression in plants. The CMS system has been used to demonstrate that numerous candidate nuclear target genes are associated with the regulation of floral organ and pollen development [15], [16], [17], [18].

Auxin plays a critical role in many processes of the plant life cycle, including embryogenesis, lateral root development, vascular differentiation, apical dominance, tropic responses and flower development (reviewed by [19]. It has long been known that auxin stimulates the transcription of primary auxin-responsive genes, which include three gene families: AUX/IAA, GH3 and small auxin-up RNA (SAUR) families [20]. Auxin is known to regulate gene expression through degradation of AUX/IAA proteins, which are degraded through the action of an ubiquitin protein called SCF^TIR1^, and auxin promotes the interaction between AUX/IAA proteins and SCF^TIR1^ [21]. In *Arabidopsis*, the ubiquitin-proteasome pathway has been shown to be involved in auxin response, based on the characterization of the auxin resistant mutants *axr1* and *tir1* [21], [22], [23]. Proteins that are destined to be destroyed are tagged with a polyubiquitin chain by a cascade reaction involving three enzymes, known as the ubiquitin activating enzyme (E1), ubiquitin conjugating enzyme (E2) and ubiquitin protein ligase (E3). Genetic evidence suggests that modification of AtCUL1 by an ubiquitin-related protein, RUB1 (related to ubiquitin 1), is essential for normal auxin response. The *Arabidopsis* RUB E2 is termed RCE (RUB-conjugating enzyme) and two RCE genes, *RCE1* and *RCE2*, were identified in the *Arabidopsis* genome [22]. The activity of the core auxin signal receptor complex SCF requires AXR1/ECR1- and RCE1-dependent modification of AtCUL1 [24].

In our previous study, the expression of the *RCE1* gene was observed to be differently expressed between MF and CMS of *Brassica juncea* by oligoarray analysis [18]. In the present study we found that over-expression of *BjRCE1* enhanced auxin response in *Arabidopsis*. We observed reduced *BjRCE1* expression and auxin response in CMS significantly; this phenotype could be mimicked by specifically inhibiting mitochondrial function. We suggested that decreased expression of *BjRCE1* might impact on the activity of CUL1 of the SCF complex and reduce auxin response in CMS.

**Figure 2:**
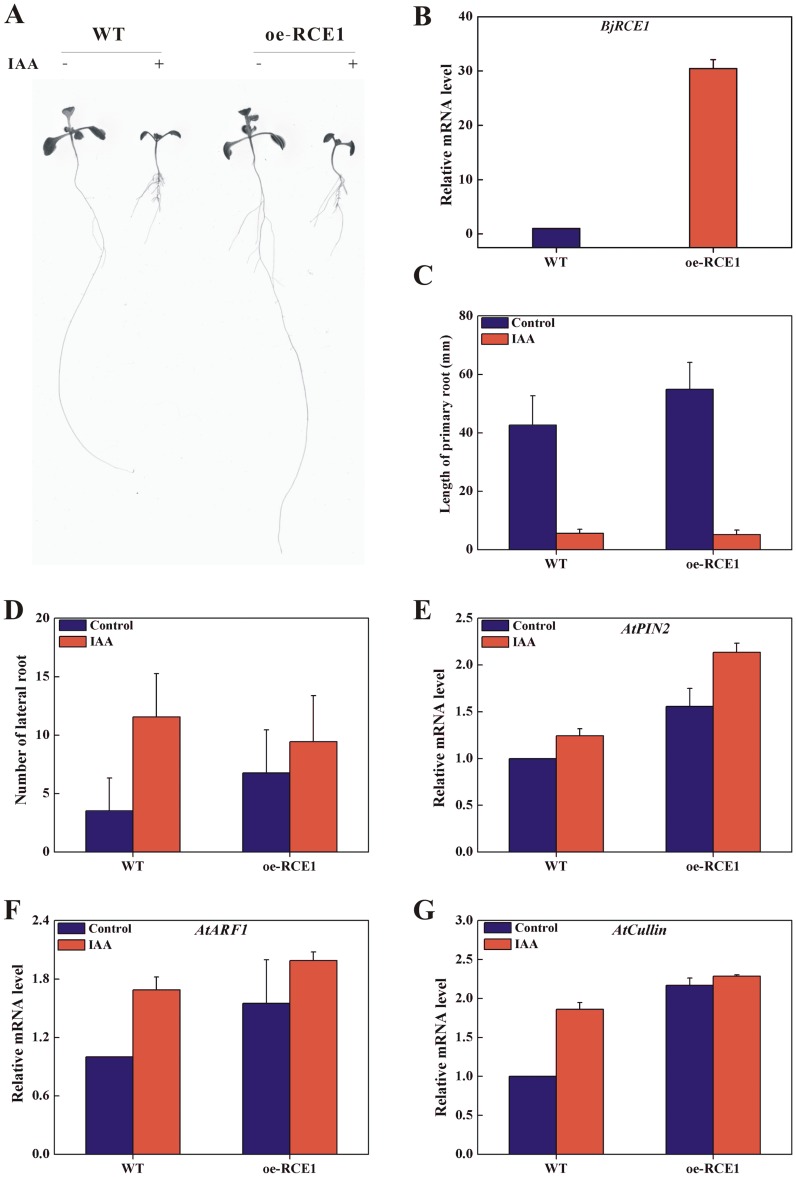
The characterization of over-expression of *BjRCE1* gene in *Arabidopsis*. A, Phenotype of oe-RCE1 of *Arabidopsis*. B, Expression level of *BjRCE1* in oe-RCE1 *Arabidopsis*. C, Statistic analysis of length of primary root. D, Statistic analysis of number of lateral root. E, Expression of *AtPIN2* gene. F, Expression of *AtARF1* gene. G, Expression of *AtCullin* gene. For genes expression, actin gene was used as an internal control. Error bars, mean±SD (three independent biological replications).

## Materials and Methods

### Plant Materials and Treatment

MF and CMS lines of *B. juncea* were developed and described in details in our laboratory [25]. The CMS *B. juncea* was developed by distant hybridization between *B. rapa* as CMS cytoplasm donor and fertile *B. juncea*, followed by repeated backcrossing with fertile *B. juncea* as recurrent parent. After backcrossing of 13 generations with fertile *B. juncea*, we got the stable CMS *B. juncea*. Meanwhile, fertile *B. juncea* was concomitantly self-crossing as its corresponding maintainer line. The progenies of the advanced backcrossed BC_13_ generation and its corresponding maintainer line were used as the sources of sterile and fertile cytoplasms, respectively. CMS and MF seeds were suspended in 0.15% (w/v) agrose and then sown onto plant MS medium. For treatment, the MS medium was supplemented with 100, 500 µM IAA separately, and 0.1 mM antimycin A (AA) (Sigma Chemical, St Louis, MO, USA) as required. Wild type (Col) and transgenic *Arabidopsis* over-expressed the *BjRCE1* gene were also suspended in 0.15% (w/v) agarose and then sown onto plant 1/2 MS medium. For treatment, the 1/2 MS medium was supplemented with 100 µM IAA.

**Figure 3:**
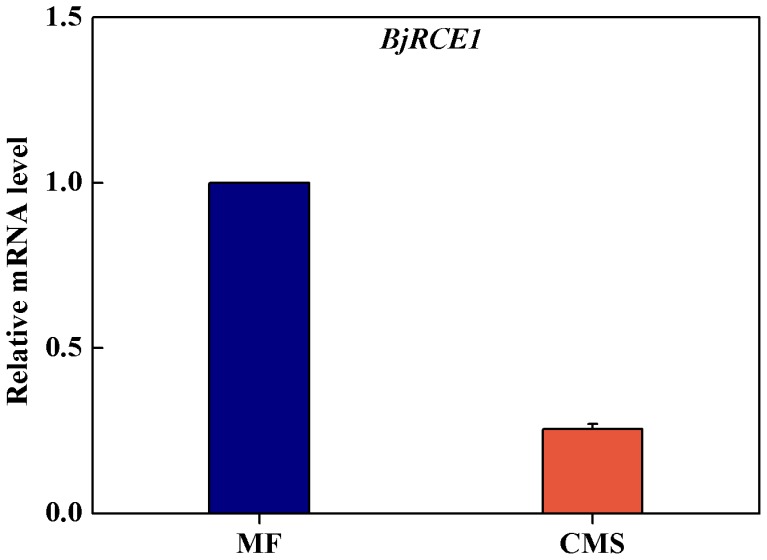
The transcriptional expression of *BjRCE1* gene in MF, CMS of *Brassica juncea*. For *BjRCE1* gene expression, 25S gene was used as an internal control. Error bars, mean±SD (three independent biological replications).

### Phenotypic Analysis of Root Development

Seedlings of CMS and MF were grown for 4 d, and seedlings of wild type and transgenic *Arabidopsis* were grown for 8 d at 28°C with 16/8 h day/night in a growth chamber. Then the root development parameters were measured by using a root scanning system (STD1600, Epson, Japan) and analysis software (Win-Rhizo, Regent Instruments, Canada).

### Isolation of *BjRCE1* Gene from *B*. *juncea*

The *RCE1* gene from *B. juncea* was homologically isolated by using reverse transcription-polymerase chain reaction (RT-PCR) combined with rapid-amplification of cDNA ends method. A cDNA fragment of *RCE1* was cloned with primers RCE1SP1 and RCE1SP2 by using RT-PCR. The primers were designed based on a sequence of *RCE1* from *Arabidopsis* (At4G36800 and At2G18600). After sequencing of this fragment, a set of anchor primers, RCE1SP3 and RCE1SP4, were designed to clone the 3′-terminal of this cDNA combined with the common primers (B25 and B26). After cloning of cDNA of *RCE1*, we sequenced the genomic structure of *RCE1* in *B. juncea*. All primers are listed in Table S1.

**Figure 4:**
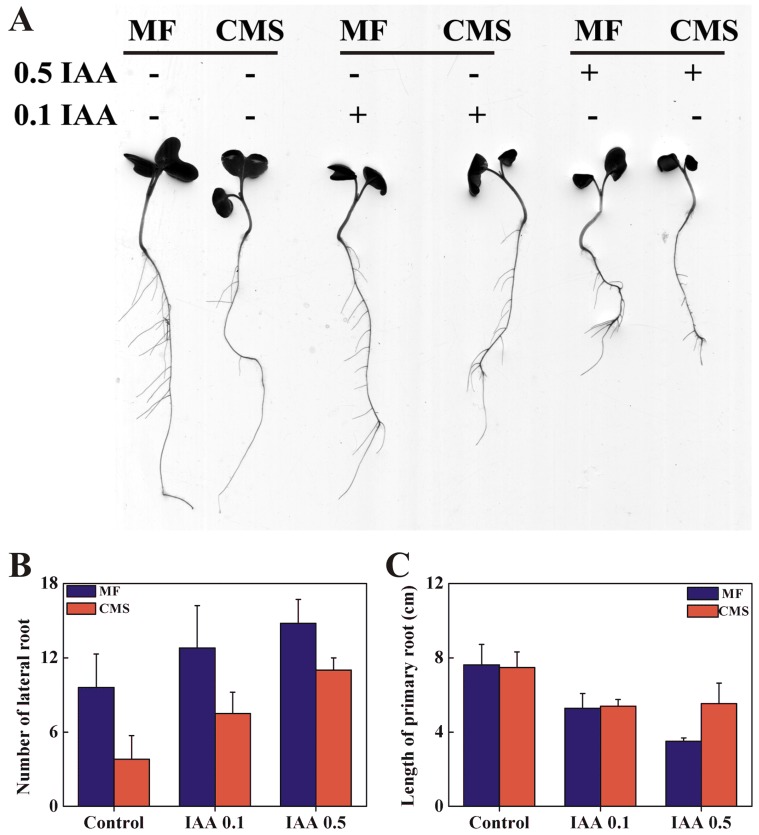
The phenotypic analysis of root from MF and CMS of *Brassica juncea*. A, Root phenotype of MF, CMS and treated with 0.1 mmol/L and 0.5 mmol/L IAA. B, Statistic analysis of lateral root number. C, Statistic analysis of primary root length. Mean±SD values from 20 seedlings.

### Construction of GFP Fusion of *BjRCE1* Gene and Transit Expression in *Arabidopsis*

The *BjRCE1* coding region was amplified using specific primers flanked by Gateway recombination cassettes (Invitrogen, California, USA). The primers used are listed in Table S1. PCR products were cloned into pDONR221 according to the manufacturer’s instructions. Cloning into the final GFP vectors (pK7FWG2) was conducted by LR reaction (Invitrogen). The mt-RFP plasmid containing the pre-sequence of *Arabidopsis thaliana* ATPase delta-prime subunit and DsRed2 was provided by Dr. S. Arimura (Laboratory of Plant Molecular Genetics, University of Tokyo) [26].

Biolistic co-transformation of the GFP and RFP fusion vectors was performed on *Arabidopsis* leaves. In brief, GFP and RFP plasmids (5 µg each) were co-precipitated onto gold particles and transformed using a PDS-100/He biolistic transformation system (Bio-Rad, www.bio-rad.com). Healthy *Arabidopsis* leaves were placed on MS medium and bombarded. Leaves were then incubated for 48 h at 22°C before microscopy using a Nikon fluorescence microscope system.

### Over-expression of *BjRCE1* Gene in Transgenic *Arabidopsis*

The amplification of *BjRCE1* coding sequences by Gateway recombination cassettes (Invitrogen) were cloned into pDONR221 according to the manufacturer’s instructions. Cloning into the final binary vectors (pK7WG2) was conducted by LR reaction (Invitrogen). Then, the pK7WG2 construction was transferred into *Agrobacterium tumefaciens* strain GV3101 and transformed into *Arabidopsis* [27]. Transgenic *Arabidopsis* over-expressed*BjRCE1* was screened by adding 20 mg/L kanamycin in 1/2 MS mediumfor two generations and PCR checking of the existence of alien *BjRCE1*gene. Then we checked the expression of *BjRCE1*in wild type and transgenic *Arabidopsis* by specific primers of *BjRCE1* gene using qPCR method.

**Figure 5:**
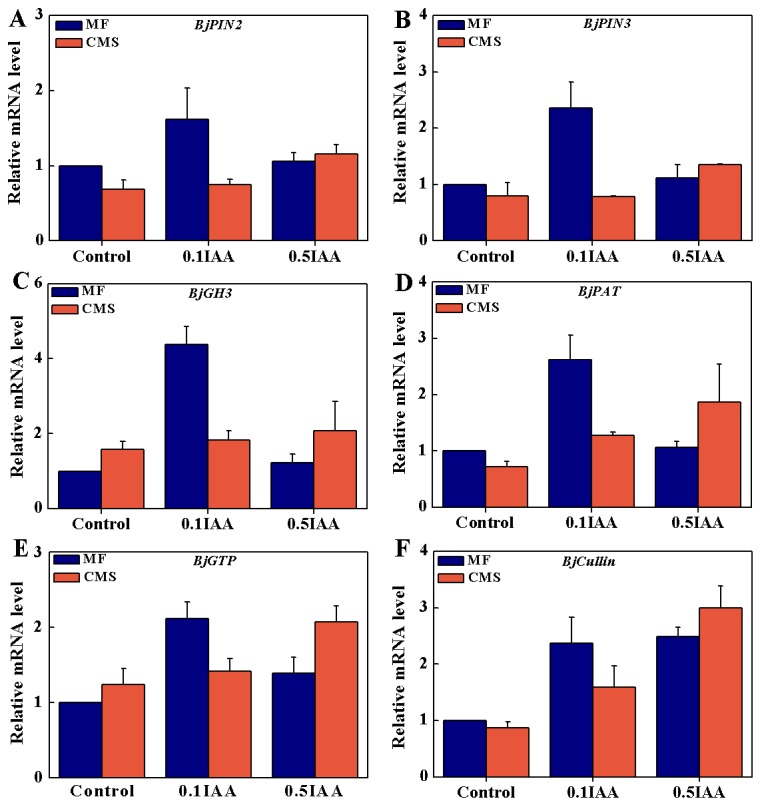
Transcriptional expression patterns of auxin-related genes in MF, CMS and MF/CMS treated with IAA in *Brassica juncea*. For genes expression, 25 S gene was used as an internal control. Error bars, mean±SD (three independent biological replications).

### RNA Extraction, Reverse Transcription and Real-time Quantitative PCR

Total RNA was extracted from seedlings using an RNeasy Plant Mini Kit (Qiagen, Valencia, CA, USA) and β-mercaptoethanol (Sigma) following the manufacturer’s protocol. During extraction, total RNA was exhaustively treated with RNase-Free Dnase (Qiagen, Germany). RNA concentration and quality were determined with a biophotometer (Eppendorf, Hamburg, Germany) and gel analysis. 1 µgtotal RNAs were transcribed to synthesize the cDNA first chain using a Reverse Transcriptase M-MLV Kit (Takara, Japan). Real-time PCR reactions were performed according to a previously established method [28]. Real-Time PCR reactions were performed using 2.5 µl of each cDNA sample, 6.5 µl of the Fast start universal SYBR Green Master (Roche Germany), and 2 µM of each primer, in a total volume of 20 µl. The ABI StepOneTM PCR System (Applied Biosystems, CA, USA) was used to detect amplification products. RT-PCR condition was as follows: 20 seconds at 95°C, followed by 40 cycles of 3 seconds at 95°C and 30 seconds at 60°C. All reactions were run in triplicate on each 48-well plate and independent experiments were repeated at least three times. The relative quantification of the target gene was determined using the ΔΔCT method. The Ct (threshold cycle) values of the target genes were normalized to the reference gene: ΔCT = Ct_target gene_–Ct_reference gene_ and compared with a calibrator (wild type): ΔΔCT = ΔCt_test Sample_–ΔCt_wild-type sample_. Relative expression RQ was calculated using the formula RQ = 2^−ΔΔCT^. We used five gradient concentration cDNA (2×dilute) as templates, made standard curve for each primer, and make sure each standard curve amplification efficiency  = 90−110%, R^2^ = 0.998−0.999. Primers used are listed in Table S2.

## Results

### Characterization of *BjRCE1* Gene of *B*. *juncea*

An homological cloning method was employed to isolate *RCE1* from *B. juncea*. Finally, we got a 558-bp-sized orf, which was assumed to encode 185 amino acids, including five exons and four introns according to comparison of cDNA and genomic sequencing of *RCE1* (Figure 1A). Bioinformatic analysis indicated the presence of a UBCc superfamily domain in *BjRCE1* gene, suggesting that its function was related to ubiquitin (Figure 1B). Alignment by Clustal W revealed that putative amino acids of RCE1 from *B. juncea* had 94 and 83% similarity with that from AtRCE1 (AT4G36800) and AtRCE1 (AT2G36800) (Figure 1C). A phylogenetic tree was constructed, based on the deduced amino acid sequences, to inspect the genetic relationships among the genes from *B. juncea* and other members of the RCE1 family. The RCE1 from *B. juncea* had close relationship with AtRCE1 (AT4G36800) from *Arabidopsis* (Figure 1E). The *RCE1* ortholog from B. *juncea* was named *BjRCE1* (NCBI No. FJ189480). Moreover, *BjRCE1* was targeted to the nucleus as shown by the GFP fusion protein fluorescence (Figure 1D).

### Over-expression of *BjRCE1* Gene Enhanced Auxin Response in *Arabidopsis*

In *Arabidopsis*, the related-to-ubiquitin (RUB) modification of CUL1 is required for normal function of the SCF^TIR1^ complex and the RCE1 protein functioned as a RUB-conjugated enzyme *in vivo*. A mutation in *RCE1* reduced auxin response and affected root development (Dharmasiri *et al*., 2003). In the present study, the over-expressed *BjRCE1* in *Arabidopsis* (oe-BjRCE1) resulted in longer primary roots and more lateral roots under normal growth conditions, and shorter primary roots and less lateral roots under IAA treatment (Figure 2A–D). The expressions of several auxin-related genes - auxin efflux carrier (*PIN2*), auxin response factor (*ARF1*) and subunit of SCF complex (*Cullin*) genes - were induced in oe-BjRCE1 *Arabidopsis* under normal and IAA treatment conditions (Figure 2E–G).

### Reduced *BjRCE1* Gene Expression and Auxin Response in CMS

Previously, the expression of *RCE1* was found to be differently expressed between CMS and MF using oligoarray analysis (Yang *et al*., 2010). After the cloning of *RCE1* from *B. juncea*, the expression of *BjRCE1* was investigated in MF and CMS by using qRCR method. There was reduced *BjRCE1* expression in CMS compared to MF (Figure 3). The number of lateral roots was significantly decreased in CMS compared to MF under normal growth conditions (Figure 4A and B). After IAA treatment, the number of lateral roots increased in both CMS and MF; however, the number of lateral roots was still less in CMS than in MF (Figure 4A and B). Primary roots were of similar lengths for CMS and MF under normal and 100 µM IAA treatments; however, primary roots were longer in CMS than in MF under 500 µM IAA treatment (Figure 4C).

To determine whether mitochondrial function could alter the auxin response, we studied the phenotype in MF treated with a specific mitochondrial inhibitor (AA). The number of lateral roots of MF treated with AA was clearly reduced compared to MF (Figure S2-A, B). Treatment with IAA and AA in MF led to an increased number of lateral roots; however, there were less lateral roots in MF treated with AA than without AA (Figure S2-A, B). The length of primary roots was also decreased in MF when treated with AA; however, primary root length was even shorter in MF treated with AA following 100 and 500 µM IAA treatments (Figure S2-C). Furthermore, the expression of *BjRCE1* in MF treated with AA was obviously reduced compared to MF plants (Figure S1).

### Expression Patterns of Auxin-related Genes in MF, CMS

We studied the expressions of auxin-related genes including auxin efflux carrier (*PIN2* and *PIN3*), auxin-responsive GH3 family protein (*GH3*), efflux carrier of polar auxin transport (*PAT*), ARF-like small GTPase (*GTP*) and subunit of SCF complex (*Cullin*) in MF, CMS and MF/CMS treated with IAA of *B. juncea*. The expressions of *PIN2*, *PIN3*, *PAT* and *Cullin* genes were decreased in CMS, and IAA treatment induced expressions of these genes in MF and CMS (Figure 5-A, B, D, F). The expressions of *GH3* and *GTP* genes were increased in CMS, and IAA treatment induced expressions of these genes in MF and CMS (Figure 5-C, E). The expression levels of all investigated genes but *Cullin* gene were higher in MF treated with 100 µM IAA compared to500 µM IAA. The expression levels of all investigated genes were higher in CMS treated with 500 µM IAA compared to 100 µM IAA (Figure 5). We also checked the expressions of these genes in MF treated with IAA and AA of *B. juncea* to study these genes expressions when mitochondrial functions were inhibited. The expressions of *PIN2*, *PIN3*, *PAT* and *Cullin* genes were decreased in MF treated with AA as CMS (Figure S3-A, B, D, F). The expressions of *GH3* and *GTP* genes were increased in MF treated with AA as CMS (Figure S3-C, E). And only 500 µM IAA treatment induced expressions of these genes in MF treated with AA (Figure S3).

**Figure 6:**
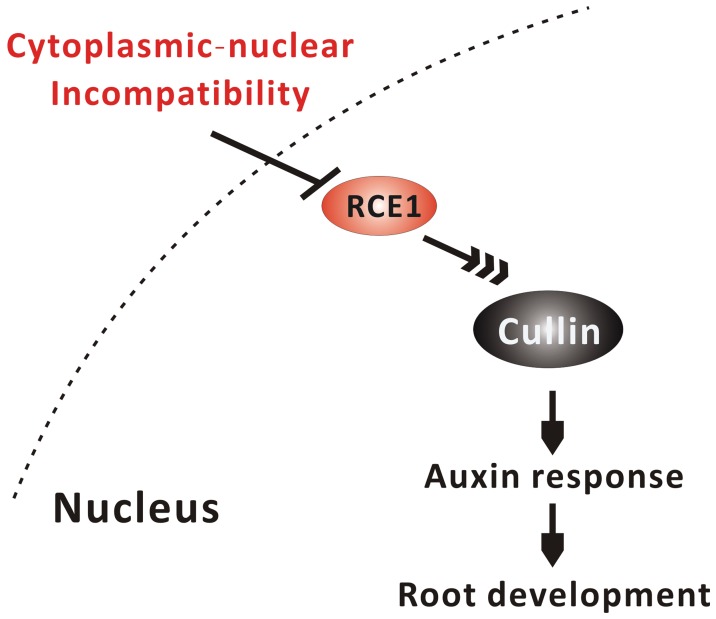
A proposed model of mitochondrial modulation of auxin response that regulates root development via *BjREC1* gene.

## Discussion

The coordination of organellar functions requires dynamic adjustment of gene expression by retrograde regulation, in which organellar stimuli modulate nuclear-encoded genes [8], [9]. Retrograde regulation is essential as the nucleus encodes the majority of organellar proteins and therefore initially controls most aspects of organellar biogenesis and function. Due to the multitude of organellar functions, a variety of interlinked retrograde pathways can be expected, however, whether the expected signals could be integrated into common pathway is still not clear.

Important progress has been made towards understanding PRR in plants, in which the GUN1 gene integrated the multiple indicators in plastid and led to ABI4-mediated the repression of nuclear gene expression [6]. However, only little is known about the MRR in plants [9]. The general process of MRR is conserved among yeast, mammals and plants; however, the mechanisms of signal transduction pathways and key signal molecules are probably diverse [8]. Up to now, at least three kinds of MRR pathways and mechanisms have been described in yeast [8], [29]. Although the MRR pathway has not been well documented in plants, compelling evidence suggests that there are multiple types of mitochondrial signaling pathways in plants [30], [31], [32], [33]. These included the observation that citrate treatment (which is assumed to affect mitochondrial function) induced alternative oxidase (AOX) gene expression but did not cause reactive oxygen species (ROS) increases in cultured tobacco and soybean cells [30], [34]. In soybean cells, this induction was blocked by a protein kinase inhibitor, but was induced by AA [30]. In *Arabidopsis*, candidate MRR mutants were screened and identified in response to distinct mitochondrial perturbations of inhibitions of the tricarboxylic acidcycle and mitochondrial electron transport chain by using the promoter of the *AOX1a* gene as a mitochondrial marker [33]. Inhibition of mitochondrial ATP synthase caused increased respiration and induced *AOX1a* expression in *Arabidopsis*, which suggested different MRR signaling pathways respectively for AA- and mtROS-induced MRR [35]. In maize CMS with mutations in different mitochondrial genes encode distinct *AOX* genes, and similar responses were seen with inhibitors of respiratory complexes [31]. Candidate nuclear target genes regulated by mitochondria caused the failure of pollen development and CMS phenotypes in several CMS systems [16], [17], [36]. MRR can also occur during heat stress, strongly inducing heat-shock-protein gene expression, whereas AA and monofluoroacetate (MFA) do not induce expression of these genes [37], [38].

We employed CMS of *B. juncea* to explore candidate retrograde regulation targets and pathways caused by the nuclear-cytoplasmic incompatibility. Previously, we identified candidate nuclear target genes that were probably regulated by the nuclear-cytoplasmic incompatibility through comparisons of gene expression in CMS and MF using oligoarray analysis [18]. In the present study, we demonstrated that expression of *BjRCE1*, one candidate retrograde regulating gene, was down-regulated in CMS. Interestingly, the expression pattern of *BjRCE1* was mimicked in MF when we specifically inhibited the mitochondrial function using AA. Indeed, the expression of *BjRCE1* was really regulated by mitochondrial dysfunction in CMS and MF treated with AA.

We also investigated that several other nuclear genes were subject to the nuclear-cytoplasmic incompatibility in CMS *B. juncea*, of which the *CTR1-like* gene altered ethylene response in CMS [28] and the *mtHSC70* gene affected temperature responses in CMS (our unpublished data). Bioinformatic analysis of CTR1-like and mtHSC70 showed ATP-binding domains within these proteins (data not shown). Meanwhile, RCE1 protein, as ubiquitin E2, functioned in an ATP-dependent process [39], [40]. In CMS *B. juncea*, the activity of mitochondrial ATP synthesis and ATP content were significantly decreased compared to MF [41]. ATP regulation of the expression of ATP-binding genes has been described in several cases: including ATP-binding cassette (ABC) transporter (Rea, 2007), heat shock protein [42], [43] and general regulator factor [43]. We concluded that mitochondria may modulate such a type of nuclear gene expression in an ATP-dependent manner, which might be one mechanism of retrograde regulation of nuclear gene expression in plants.

In *Arabidopsis*, RCE1 is required for RUB (related-to-ubiquitin) modification of the Cullin subunit of the SCF complex function as RUB-E2. The *Arabidopsisrce1* mutant is deficient in auxin and jasmonate responses [44]. In the present study, we confirmed the relationship between *BjRCE1* and auxin response in *Arabidopsis* over-expressed*BjRCE1*. Because of decreased expression of *BjRCE1* in CMS and MF treated with AA, the auxin response was subsequently reduced in terms of root development and auxin-related gene expression. We also observed altered jasmonate response in CMS (data not shown). Importantly, the phenotype of the reduced auxin response was mimicked in MF when we specifically inhibited mitochondrial function using AA. This indicated mitochondria modulated auxin response via *BjRCE1* in CMS *B. juncea*. Recent studies have reported that the *ABI4*, encoding a member of the DREB subfamily A-3 of the ERF/AP2 transcription factor and which was ever identified as a target gene of chloroplast retrograde regulation, also played an important role in mediating MRR signals to induce the expression of *AOX1a* in *Arabidopsis* [45]. This means that mitochondria can retrograde modulate ABA response via *ABI4*. In a previous study, we studied retrograde regulation of ethylene response via the *CTR-like* gene in CMS *B. juncea* [28]. If this is so, we can modulate mitochondrial function to regulate the corresponding nuclear gene expression and biological traits, and then utilize this in crop breeding strategies.

In conclusion, our results established a link between retrograde regulation of *BjRCE1* expression and the auxin signal pathway regulating root development in CMS *B. juncea*. The results led us to propose that decreased expression of *BjRCE1* may impact on CUL1 of the SCF complex and reduce auxin response in CMS (Figure 6). How *BjRCE1* or the ubiquitin cascade pathway can sense signals from organelle remains to be investigated in further studies.

## Supporting Information

Figure S1 The transcriptional expression of *BjRCE1* gene in MF, CMS and MF/CMS treated with AA in *Brassica juncea*. For *BjRCE1* gene expression, 25 S gene was used as an internal control. Error bars, mean ± SD (three independent biological replications).
(TIF)

Figure S2 The phenotypic analysis of root from MF and MF treated with AA and IAA in*Brassica juncea*. A, Root phenotype of MF, and treated with 0.1 mmol/L, 0.5 mmol/L IAA and 0.5 mmol/L AA. B, Statistic analysis of lateral root number. C, Statistic analysis of primary root length. Mean ± SE values from 20 seedlings.
(TIF)

Figure S3 Transcriptional expression patterns of auxin-related genes in MF and MF treated with AA and IAA in *Brassica juncea*. For genes expression, 25 S gene was used as an internal control. Error bars, mean ± SD (three independent biological replications).
(TIF)

Table S1 Primers of cloning and localization of *BjRCE1* gene were listed as followings.
(DOC)

Table S2 Q-PCR primers used in this study were listed as followings.
(DOCX)
