# Supplementary figures and images for: Reduced Expression of BjRCE1 Gene Modulated by Nuclear-Cytoplasmic Incompatibility Alters Auxin Response in Cytoplasmic Male-Sterile Brassica juncea
Source: PLoS One. 2012 Jun 18;7(6):e38821. doi: 10.1371/journal.pone.0038821 (PMC3377708; doi:10.1371/journal.pone.0038821)

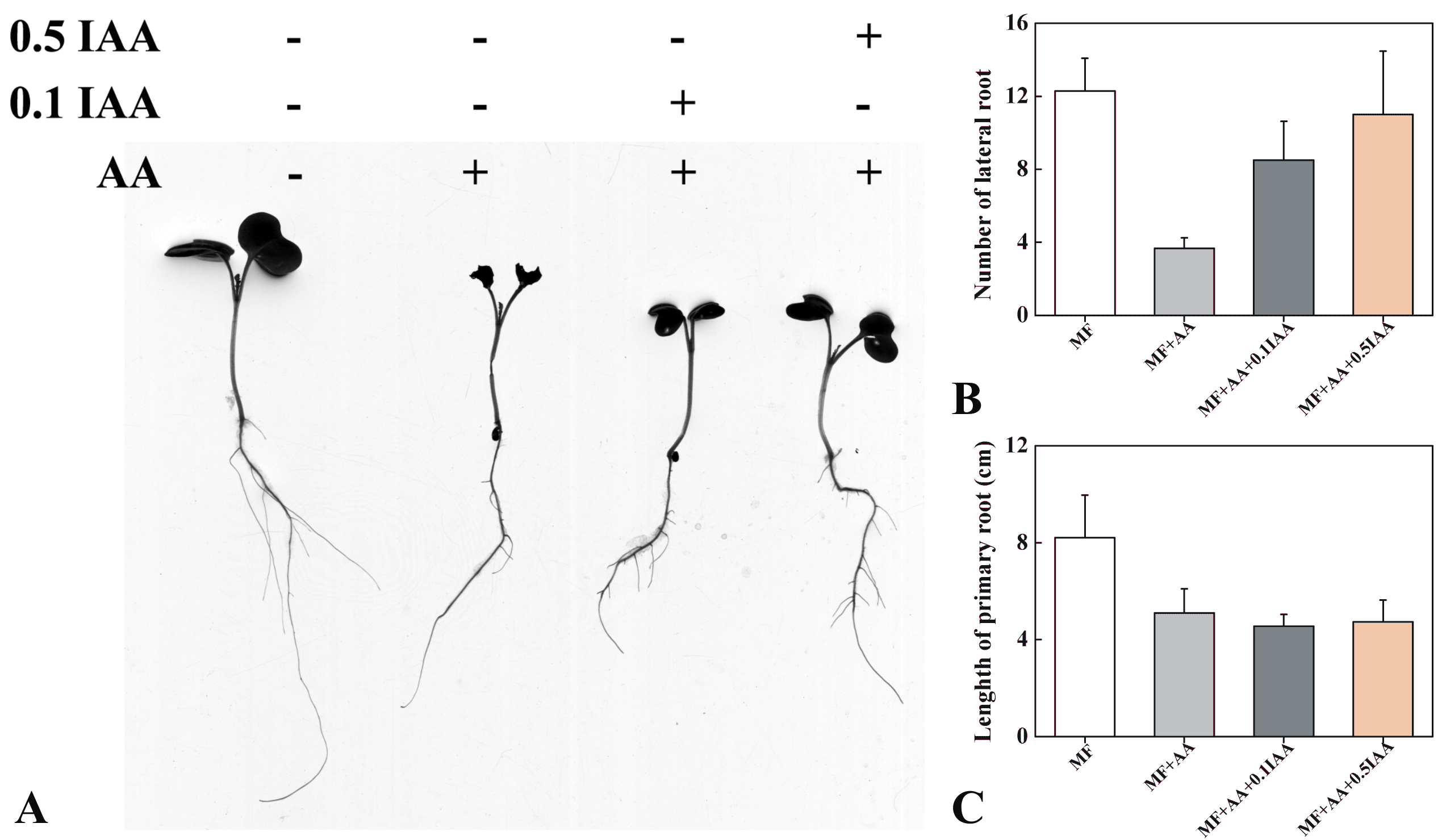

Supplement: Figure S2 — The phenotypic analysis of root from MF and MF treated with AA and IAA inBrassica juncea. A, Root phenotype of MF, and treated with 0.1 mmol/L, 0.5 mmol/L IAA and 0.5 mmol/L AA. B, Statistic analysis of lateral root number. C, Statistic analysis of primary root length. Mean ± SE values from 20 seedlings. (TIF) [file pone.0038821.s002.tif]

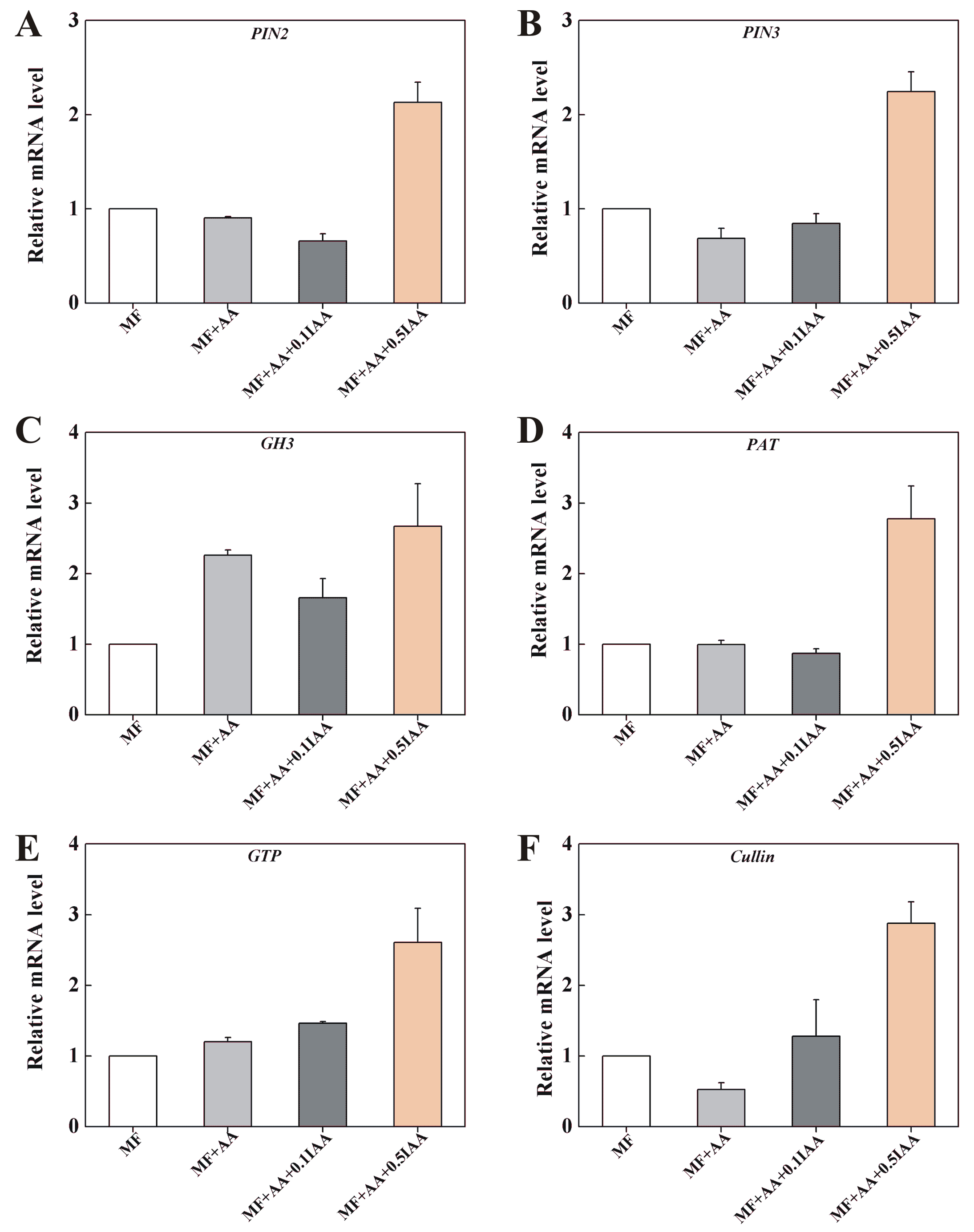

Supplement: Figure S3 — Transcriptional expression patterns of auxin-related genes in MF and MF treated with AA and IAA in Brassica juncea. For genes expression, 25 S gene was used as an internal control. Error bars, mean ± SD (three independent biological replications). (TIF) [file pone.0038821.s003.tif]
