# Supplementary material for: Reduced Expression of BjRCE1 Gene Modulated by Nuclear-Cytoplasmic Incompatibility Alters Auxin Response in Cytoplasmic Male-Sterile Brassica juncea
Source: PLoS One. 2012 Jun 18;7(6):e38821. doi: 10.1371/journal.pone.0038821 (PMC3377708; doi:10.1371/journal.pone.0038821)
Supplement: Table S1 — Primers of cloning and localization of BjRCE1 gene were listed as followings. (DOC) [file pone.0038821.s004.doc]

Table S1 Primers of cloning and localization of *BjRCE1* gene were listed as followings.

| Primer name | Sequences |
| --- | --- |
| RCE1SP1 | ATGATTGGGTTGTTTAAAGT |
| RCE1SP2 | TCA A/G GCA C/A AC A/G TTTCCTTCCA |
| GFP-BjRCE1-F | CACCATGATTGGGTTGTTTAAAGT |
| GFP-BjRCE1-R | GATACAGCGAGGGAAGTTGGG |
| OE-BjRCE1-F | CACCATGATTGGGTTGTTTAAAGT |
| OE-BjRCE1-F | TTAGATACAGCGAGGGAAGTTGGG |
