# Supplementary material for: Reduced Expression of BjRCE1 Gene Modulated by Nuclear-Cytoplasmic Incompatibility Alters Auxin Response in Cytoplasmic Male-Sterile Brassica juncea
Source: PLoS One. 2012 Jun 18;7(6):e38821. doi: 10.1371/journal.pone.0038821 (PMC3377708; doi:10.1371/journal.pone.0038821)
Supplement: Table S2 — Q-PCR primers used in this study were listed as followings. (DOCX) [file pone.0038821.s005.docx]

Table S2 Q-PCR primers used in this study were listed as followings.

| Gene | Forward primer | Reverse primer |
| --- | --- | --- |
| *BjRCE1* | AGGAGGAGGAGCTTCTGTCA | TTCATCAAATCATCTTTGCCA |
| *BjPIN2-2* | CATTGGATTGCATGGTGATT | CAAAGGGTACAATCCCTTGAG |
| *BjPIN3-1*  *BjGH3-1* | CACTGCCTCAAGGGATTGTA  GGAAGACATGAAGGAGCTTGA | ATCGGAAGCGCTATAAGCAT  GGCCCTATAAATTCGTTAGCC |
| *BjPAT-1* | CCTCAAGGGATTGTACCGTT | AAACCAGCGTGATAGGAAGC |
| *BjGTP-1* | ACAAGCTTGGCCTTCACTCT | TTGTTGGAGAGCCAGTCAAG |
| *Bj25S* | CGGTTCCTCTCGTACTAGGTTGA | CCGTCGTGAGACAGGTTAGTTTT |
| *BjCullin* | TGGACCAAGGATGGGACT | GGCTTGAACATTCGGCTA |
| *AtPIN2* | TAAGGAATGCTGATGCCAAC | CTCCTTATCTGCGTCCCATT |
| *AtARF* | GGGATGCAGTTCTGCTTGTA | GAAGAGAATGAAGCCCAAGC |
| *AtCullin* | GCCTGATATCAAAGCGATCA | ATTCCTTTCATGAGCCAACC |
| *AtActin* | TATCGCTGACCGTATGAGCAAAG | TGGACCTGCCTCATCATACTCG |
